# Perceived Opportunities to Craft Scale: adaptation and evidence of the construct validity of the Brazilian version

**DOI:** 10.1186/s41155-020-00158-5

**Published:** 2020-09-07

**Authors:** Rita Pimenta de Devotto, Clarissa Pinto Pizarro de Freitas, Solange Muglia Wechsler

**Affiliations:** 1 Faculdades de Campinas (Facamp), Avenida Alan Turing, 805, Cidade Universitária, Campinas, São Paulo, 13083-898 Brazil; 2 Pontifícia Universidade Católica de Campinas (PUCCAMP), Avenida Alan Turing, 805, Cidade Universitária, Campinas, Campinas, Brazil; 3 Pontifícia Universidade Católica do Rio de Janeiro (PUC-Rio), Rio de Janeiro, Brazil

**Keywords:** Perceived opportunity to craft, Job crafting, Occupational self-efficacy, Work engagement, Workaholism, Validation

## Abstract

Employees’ perceived opportunities to craft (POCs) influence actual job crafting behavior, which may have consequences for their well-being and work performance. This study aimed to validate the *perceived opportunities to craft scale* (POCS) in the Brazilian context. We collected data from Brazilian employees (*N* = 1451) in two separate samples. The factor structure, reliability, and convergent validity of the POCS were tested using confirmatory factor analysis (CFA), multigroup confirmatory factor analysis (MGCFA), and external variables. The results indicated that the POCS-Brazilian version (with seven-point and five-point Likert scale) replicated the unidimensional structure of the original instrument. POCs showed significant positive and moderate correlations with occupational self-efficacy, work engagement and job crafting, and negative associations with workaholism. Both POCS-Brazilian versions were reliable according to three types of reliability indexes and also showed discriminant and convergent validity evidence.

The emergence of the Fourth Industrial Revolution and the types of challenges caused by the VUCA environment (i.e., volatility, uncertainty, complexity, and ambiguity) in which organizations operate (Sathyanarayan & Lavanya, 2018) are transforming jobs around the world and requiring more flexible and favorable work designs. Job profiles became more about responsibilities and delivered results and less about predefined tasks (van Wingerden & Poell, 2017). Due to the increased uncertainty and dynamism in organizations, management must count on employees taking initiative to anticipate and create changes in how work is performed (Grant & Parker, 2009).

Proactive perspectives on work design, specifically research about job crafting, emphasized the importance of employees aligning their changing jobs and their own strengths, values, and passions (Berg, Dutton, & Wrzesniewski, 2008; Wrzesniewski & Dutton, 2001). The job crafting literature also called attention to employees’ continuous efforts to optimize the levels of their job demands and job resources with personal resources (Tims & Bakker, 2010; Tims, Bakker, & Derks, 2012). Since the publication of the seminal article of Wrzesniewski and Dutton in Wrzesniewski & Dutton, 2001, job crafting became a prominent topic and the research has grown rapidly (Zhang & Parker, 2019).

Although job crafting is a creative and improvised process that focuses primarily on improving the person-job fit (Wrzesniewski & Dutton, 2001), there is evidence that job crafting behavior predicts positive well-being outcomes and improves individual work performance. Job crafting has been found to be positively associated with increased work engagement (Pimenta de Devotto et al. 2020), job performance (van Wingerden, Bakker, & Derks, 2016), and well-being indicators (Slemp & Vella-Brodrick, 2013). As a result of this evidence, job crafting has also become a subject of interest among human resource practitioners and senior management.

Different work contexts may enable or hinder different levels and forms of job crafting (Wrzesniewski & Dutton, 2001). Employees’ actual job crafting behavior is affected by their perceived opportunities to craft (POCs), which in turn, are influenced by their work environment characteristics (e.g., availability of job resources), by management’s top down job design initiatives, and by internal factors (e.g., personal resources). In situations where the POCs are low, the perception of not having opportunities to change tasks and interactions may inhibit job crafting behavior (van Wingerden & Niks, 2017). Thus, as POCs are a precondition for actual job crafting behavior, insights into employees’ POCs may contribute to assessing and increasing the impact of job crafting behavior in organizations.

Based on this information, this study examined the psychometric properties (i.e., reliability, factorial validity, and measurement invariance) of the *perceived opportunity to craft scale* (POCS) (van Wingerden & Niks, 2017) in the Brazilian context. After that, we cross-validated the factor structure of the POCS and examined its evidence based on its relations with external variables. We aim to advance the research on job crafting and encourage more empirical research on the POCS, especially in Brazil.

## Job crafting conceptualization

Job crafting was originally defined as “the physical and cognitive changes individuals make in the tasks or relational boundaries of their work” (Wrzesniewski & Dutton, 2001, p.179). Accordingly, job crafting encompasses three types of strategies: (1) task crafting (e.g., actual alterations to the number or scope of tasks), (2) cognitive crafting (e.g., reframing the meaning of and identity at work), and (3) relational crafting (e.g., changes in the quantity and quality of workplace relationships) (Wrzesniewski & Dutton, 2001). Thus, the seminal theoretical perspective on job crafting encompasses self-initiated, cognitive, and behavioral changes in a job. Task and relational crafting produce actual changes in the job characteristics and the social work environment, whereas cognitive crafting relates to the intangible (mental) changes about the perceptions of work’s meaning and purpose (Lichtenthaler & Fischbach, 2016).

Since job crafting entails tangible changes in the job demands and job resources, Tims and Bakker (2010) proposed its integration into the job demand–resources (JD-R) theory (Bakker & Demerouti, 2017). Within the JD-R perspective, job crafting is defined as self-initiated changes to optimize the levels of job resources and job demands with personal abilities and needs (Tims & Bakker, 2010; Tims et al., 2012). In this theoretical perspective, job crafting was operationalized according to the types of job resources and job demand characteristics suggested by the JD-R theory. The *job crafting scale* developed by Tims et al. (2012) consists of four dimensions: increasing social job resources (e.g., looking for peer and supervisor feedback), increasing structural job resources (e.g., increasing job autonomy and intensifying the opportunities for self-development), increasing challenging job demands (e.g., taking on new responsibilities), and decreasing hindering job demands (e.g., diminishing the number of tasks).

These two distinct theoretical perspectives on job crafting are quite similar since they emphasize an employee’s self-initiated changes to expand and enrich their jobs and roles, or to reduce and limit them (Zhang & Parker, 2019). A major difference among both job crafting perspectives is that while Tims et al. (2012) defined job crafting as tangible behavior based on changes in job characteristics, Wrzesniewski and Dutton (2001) considered a cognitive dimension in which employees can modify their perceptions and views about their work. The cognitive component of job crafting is the dimension that aligns most closely to the changes in work identity and the meaning of work (Berg et al., 2008).

Recently, theoretical integrative efforts synthesized eight defining characteristics of job crafting: (a) it is self-targeted to benefit the agent; (b) it is conscious and intentional change, (c) it produces a noticeable deviation between the crafted and the real job; (d) it produces permanent or semipermanent changes; (e) it changes the job role, not the leisure activities; (f) it applies to a job with a clear job description; (g) it changes the intrinsic rather than extrinsic job characteristics (e.g., pay); and (h) it does not require formal approval from a superior (Bruning & Campion, 2018; Zhang & Parker, 2019).

Zhang and Parker (2019) proposed a three-level hierarchical structure of job crafting to integrate the two theoretical perspectives—the seminal theory from Wrzesniewski and Dutton (2001) and the JD-R perspective from Tims et al. (2012)—and advance the research. The highest level—*job crafting orientation*—distinguishes whether crafting is approach oriented (i.e., effortful and directed actions to seek the positive aspects of work) or avoidance oriented (i.e., effortful and directed actions to move away from the negative aspects of work). The second level—*job crafting form*—differentiates whether crafting is behavioral or cognitive. The third level—*job crafting content*—discriminates between the targets that crafting actions seek to change and whether they are primarily job resources or job demands.

According to the *job crafting content* level (Zhang & Parker, 2019), eight specific types of job crafting were derived: (a) approach resources crafting (behavioral) (e.g., increasing job autonomy), (b) approach demands crafting (behavioral) (e.g., optimization of work process to deal with workload), (c) approach resources crafting (cognitive) (e.g., seeing how one’s job contributes to the community), (d) approach demands crafting (cognitive) (e.g., viewing demands as opportunities for professional development), (e) avoidance resources crafting (behavioral) (e.g., avoiding a project that lacks autonomy), (f) avoidance demands crafting (behavioral) (e.g., escape from projects with hindering demands), (g) avoidance resources crafting (cognitive) (e.g., cognitively moving away from activities with low positive resources), and (h) avoidance demands crafting (cognitive) (e.g., minimizing the demanding job aspects).

This hierarchical and multidimensional approach offers some advantages to the research on job crafting because of the following: (a) it may help to unite crafting concepts under a robust construct, (b) it can capture unexplored forms of crafting (e.g., approach demand crafting), and (c) it enables researchers to investigate the notion of avoidance crafting as a proactive behavior or cognition that protects from future negative outcomes (Zhang & Parker, 2019). Thus, job crafting encompasses the proactive changes, behavioral and cognitive, that employees make in their jobs in order to improve job resources and job demands, or reduce them (Zhang & Parker, 2019). We adopted this new conceptualization to examine the findings of our study.

### Perceived opportunities to craft

Wrzesniewski and Dutton (2001) argued that employees engage in job crafting to maintain control over their work, to create a positive self-image for themselves in their work, and to connect with others in the workplace. The POCs were suggested to moderate the relationship between an individual’s motivation to craft and job crafting behaviors. Furthermore, the POCs can restrict or create possibilities for employees to modify their jobs. POCs are influenced by internal (e.g., personal resources) and external (e.g., job resources and job demands) factors.

The POCs are psychologically positive since they “refers to the sense of freedom or discretion employees have in what they do in their job and how they do it” (Wrzesniewski & Dutton, 2001, p. 183). Lately, POCs have been defined as employees’ perceptions regarding their opportunities to proactively optimize their work environment (van Wingerden & Poell, 2017). Engaged employees are expected to perceive opportunities for job crafting at work before changing the task and relational boundaries at work. POCs were found to be positively related to job crafting, job resources, and work engagement and negatively related to cynicism, which is a dimension of burnout (van Wingerden & Niks, 2017). The POCs were also found to be positively related to in-role performance through job crafting and work engagement (van Wingerden & Poell, 2017).

### Assessment of the Brazilian version of the perceived opportunities to craft scale

The POCs reflect an overall perception of the extent to which employees can influence their work environment. The *perceived opportunity to craft scale* (POCS) was recently developed to empirically examine the relation between employees’ perceived opportunities to craft and their actual job crafting behavior, as well as to help researchers gain insights into the antecedents and consequences of job crafting (van Wingerden & Niks, 2017). The POCS was developed and validated in a study conducted in the Netherlands (*N* = 2329). The results indicated that the scale is a generic, reliable instrument (*α* = 0.85) with five items and a one-dimensional structure. The POCs, measured by POCS, can be empirically distinguished from the criteria-related measures (e.g., job crafting, job autonomy, and work engagement) (van Wingerden & Niks, 2017).

Although the construct is conceptually appealing for research and practice, there was no method for empirically measuring POCs in Brazil. Therefore, the present study was designed to validate the POCS (van Wingerden & Niks, 2017) in the Brazilian context. We expect that the POCS-Brazilian version presents a one-dimensional structure (*hypothesis 1*).

Regarding the rating of the scale, the original POCS uses a seven-point Likert scale with anchors only at point 1 (totally disagree) and point 7 (totally agree). There is relatively little consensus in the literature regarding a psychometrically optimal number of response options for Likert-type rating scale, and no psychometric advantages were revealed for any response scales beyond six options (Simms, Zelazny, Williams, & Bernstein, 2019). In Brazilian psychology research, the use of seven-point Likert grading is unusual and anchor terms on all points of the scale grades are preferable for readability and simplicity (Damásio, Freitas, & Koller, 2014). Participants may have difficulty perceiving differences between similarly worded response options in a seven-point Likert scale (e.g., *strongly agree* vs. *very strongly agree*). Based on that, we also evaluated a version of the Brazilian POCS using a five-point Likert scale ranging from 1 (totally disagree) to 5 (totally agree) by providing an anchor term at each point of the scale. To evaluate which of these versions presented a more coherent gradual sequence of possible responses to the scale, the two versions of the Brazilian POCS were assessed in this study: one with the seven-point Likert scale (POCS-L7) and another with the five-point Likert scale (POCS-L5). We hypothesize that the one-factor model will show a good fit to the POCS-L5 (*hypothesis 2*).

Despite the social advances and the achievements of women in different social contexts, it is observed that female professionals still face gender inequality at work. Women dealing with gender inequality in the workplace have fewer challenging work experiences than their male counterparts and feel higher levels of unpleasantness, which may undermine their perceptions of meaningfulness at work (Qian & Fan, 2019). Research concerning gender differences in job crafting is somewhat equivocal; however, a recent meta-analysis found that women engage in job crafting (i.e., increasing structural and social job resources) to a greater extent than men (Rudolph, Katz, Lavigne, & Zacher, 2017). Job crafting represents an opportunity for women to proactively manage their career development and cope with the lack of equality at work. Besides the gender differences that stem from actual job crafting behavior levels, it is expected that women comprehend and perceive opportunities for job crafting in a similar manner than men. Building upon this, we expected that the two versions of the POCS will show measurement invariance for sex (*hypothesis* 3).

In addition, we examined the convergent validity of the POCS by relating the POCs to theoretically related constructs. First, we expected a positive relationship between POCs and job crafting (*hypothesis 4*). Following the conceptualization of job crafting as a three-level hierarchical structure (Zhang & Parker, 2019), we expect that POCs will be positively related to approach resource and approach demand crafting (behavioral and cognitive) (*hypothesis 4a*).

Second, we expect POCs to be positively related to occupational self-efficacy (*hypothesis 5*). Meta-analytical and empirical studies showed that general self-efficacy was positively related to approach crafting (Zhang & Parker, 2019). The occupational self-efficacy, a contextual form of self-efficacy, is defined as the perceptions of an individual about his abilities to effectively perform work tasks (Rigotti, Schyns, & Mohr, 2008). The occupational self-efficacy is a personal resource that influences both an employee’s perceptions of his capacity to shape the work environment and his way of coping with job demands and job resources at work.

We also investigated how POCs are related to favorable and unfavorable work-related mental states. In this sense, we examined its associations with work engagement and workaholism, which are antagonistic work-related mental states. Work engagement refers to a positive, fulfilling, affective-cognitive work-related state of mind that is characterized by vigor, dedication, and absorption (Schaufeli, Shimazu, & Taris, 2009). Work engagement is a high-activation form of positive affect (Hakanen, Peeters, & Schaufeli, 2018). Engaged employees tend to work hard (vigor), be highly involved (dedicated), and feel engrossed (absorbed) in their work (Schaufeli et al., 2009). Work engagement was found to be positively related to approach resource crafting and to approach demand crafting and negatively related to avoidance demand crafting (Zhang & Parker, 2019).

Workaholism is the tendency to work excessively hard (the behavioral dimension) and being obsessed with work (the cognitive dimension), which manifests itself in working compulsively (Schaufeli et al., 2009). Workaholism is a high-activation form of negative affect. Workaholics and engaged employees may work similarly hard; however, their underlying motivations to do so differ at their essence (Hakanen et al., 2018). Workaholics work excessively hard because of their compulsive drive; they deplete their energy and are at risk of developing burnout. Engaged workers are intrinsically motivated and enjoy working hard (Schaufeli et al., 2009).

Thus, we hypothesized that POCs are positively related to work engagement (*hypothesis 6*). Engaged employees have a positive perception of their opportunities to reshape their tasks and social work environment and use their strengths, values, and motives accordingly. In contrast, we expected POCs to be negatively associated with workaholism. Workaholic employees work excessively and compulsively, which may negatively influence their perceived opportunities to craft their jobs and align them with their personal resources (e.g., strengths, values, and passions). We therefore hypothesized a negative relationship between POCs and workaholism (*hypothesis 7*).

Finally, POCS discriminant validity evidence was investigated through the calculation of the average variance extracted (AVE) of POCs, job crafting behaviors, occupational self-efficacy, work engagement, and workaholism. The discriminating validity of the scale is observed when the AVE of each construct is superior to the square correlations of the constructs with each other (Fornell & Larcker, 1981). Based on this proposition, it was expected that square correlation of POCs with job crafting behaviors, occupational self-efficacy, work engagement, and workaholism would be lower than the AVE of each construct (*hypothesis 8*).

## Method

### Adaptation of the POCS to Brazilian Portuguese

We followed the general guidelines presented in the literature (Borsa, Damásio, & Bandeira, 2012) in which systematic steps were taken to adapt the POCS to Brazilian Portuguese. After the authors’ consent, three independent and bilingual translators translated the instrument from English to Brazilian Portuguese. The first two authors of this study synthesized the three translated versions into a preliminary adapted version. Subsequently, two bilingual specialists with experience in the field of psychological assessment and organizational psychology evaluated our synthesis with no changes suggested at this stage. A back-translated version of the POCS, which was produced by a professional translator, was approved by the author of the original instrument since it presented semantic and idiomatic equivalence to the original version of the scale. After the authors’ consent, the final version of the instrument, the *Escala de Percepção de Oportunidades para o Redesenho do Trabalho*—EPORT, was applied to a pilot group of professionals (*N* = 15) from different occupations (e.g., nurses, professors, engineers, psychologists, business) and hierarchical levels (e.g., top and middle managers and administrative staff) in order to investigate the content validity. The pilot group did not suggest any adaptations in the wording of the items and there was no inclusion of new items in the EPORT.

### Measures

*Sociodemographic Questionnaire.* This instrument was developed to assess the sociodemographic characteristics of the sample (e.g., sex, age, and educational level).

P*erceived opportunity to craft scale* (POCS; Van Wingerden & Niks, 2017). The original scale consists of five items answered on a seven-point Likert scale ranging from 1 (totally disagree) to 7 (totally agree). Given that this type of grading is less used in Brazilian psychology research, and anchor terms in each point of the scale were desirable for readability and simplicity, a version of the POCS-L5 (Appendix) ranging from 1 (totally disagree) to 5 (totally agree) was also applied.

*Occupational Self-efficacy Scale–Short form Brazilian Version* (OSS-SF; Rigotti et al., 2008, adapted by Damásio et al., 2014). It is composed of five items that are answered on a five-point Likert scale ranging from 1 (strongly disagree) to 5 (strongly agree). The OSS-SF Brazilian version presented adequate psychometric properties (*α* = .78) (Damásio et al., 2014). In the present study, the scale showed adequate psychometric properties, observed on the goodness of fit (*χ*^2^ (*gl*) = (9) 13.2, CFI = 0.990, TLI = 0.984, RMSEA (90% C.I.) = 0.040 (0.060–0.084)), and reliability indexes (α (95% C.I.) = 0.793 (0.775–0.810), ϖ (95% C.I.) = 0.720 (0.655–0.785), *cr* = 0.798).

*Job Crafting Questionnaire* (JCQ; Slemp & Vella-Brodrick, 2013, adapted by Pimenta de Devotto & Machado, 2020). This is a scale composed of 15 items, which are answered on a six-point Likert scale ranging from 1 (rarely) to 6 (very often). The scale assesses three dimensions and presented satisfactory composite reliability (*cr*) indexes (task crafting, *cr* = 0.80; cognitive crafting, *cr* = 0.93; and relational crafting, *cr* = 0.75). The psychometric properties of the scale were adequate in the present sample, as shown on the goodness of fit (*χ*^2^ (*gl*) = (62) 309.5, CFI = 0.921, TLI = 0.901, RMSEA (90% C.I.) = 0.118 (0.105–0.131)), and reliability indexes (task crafting, α (95% C.I.) = 0.734 (0.711–0.756), ϖ (95% C.I.) = 0.828 (0.784–0.871), *cr* = .800; cognitive crafting, α (95% C.I.) = 0.903 (0.895–0.911), ϖ (95% C.I.) = 0.700 (0.637–0.755), *cr* = .891; and relational crafting, α (95% C.I.) = 0.729 (0.706–0.751), ϖ (95% C.I.) = 0.684 (623–0.745), *cr* = 0.833).

*Utrecht Working Engagement Scale* (UWES-9; Schaufeli, Bakker, & Salanova, 2006, adapted by Vazquez, Magnan, Pacico, Hutz, & Schaufeli, 2015). It consists of nine items, which are answered on a seven-point Likert scale ranging from 0 (never) to 6 (always). The UWES-9 Brazilian version showed good psychometric properties (*α* = 0.94). In the sample, analyzed scale showed adequate psychometric properties, observed on the goodness of fit (*χ*^2^ (*gl*) = (27) 206.8, CFI = .953, TLI = 0.937, RMSEA (90% C.I.) = 0.097 (0.077–0.118)), and reliability indexes (α (95% C.I.) = 0.895 (0.886–0.904), ϖ (95% C.I.) = 0.891 (0.870–0.914), *cr* = 0.918).

*Dutch Work Addiction Scale* (DUWAS, Schaufeli, Taris & Bakker, 2008, adapted by Vazquez et al., 2017). It consists of 10 items answered on a four-point Likert scale ranging from 1 (almost never) to 4 (almost always). The DUWAS-10 Brazilian version showed good psychometric properties (*α* = .80). The DUWAS showed satisfactory psychometric properties in the present study, as observed on the goodness of fit (*χ*^2^ (*gl*) = (35) 176.5, CFI = 0.942, TLI = 0.925, RMSEA (90% C.I.) = 0.100 (0.082–0.118)), and reliability indexes (α (95% C.I.) = 0.842 (0.828–0.855), ϖ (95% C.I.) = 0.799 (0.763–0.835), *cr* = 0.855).

### Procedures

#### Data collection

The participants were recruited using a convenience sampling technique. Various sources, such as social and professional media networks (e.g., Facebook, LinkedIn) and the HR departments of institutions were used. Overall, 73% of the total sample that answered the POCS-L7 responded to it and the other questionnaires on a web-based platform (i.e., SurveyMonkey), whereas the remaining 27% answered the instruments in the paper-and-pencil form. The paper-and-pencil data collection form was performed at the participants’ workplace, during working hours or breaks by a member of the research team. All the participants that answered the POCS-L5 responded to it and the other questionnaires on a web-based platform (i.e., SurveyMonkey).

### Participants

Two samples were used to investigate the validity of the dimensionality of the POCS-Brazilian version. In the first sample, 1092 employees filled out the survey with the POCS-L7. The majority of the sample was female (54%), and the mean age of the participants was 38 years (SD = 9.3). Most participants (87%) held a postgraduate degree: 52% had a PhD degree, 15% had a master’s degree, and 10% had a specialization course. Most participants had worked at the same job for more than 10 years (50%), followed by professionals who had worked between 5 and 10 years (17%). The majority of participants worked in education services (66%).

In the second sample, 359 employees answered the POCS-L5. The majority of the sample was female (62%), and the mean age of the participants was 45 years (SD = 14.6). Overall, 58% of participants held a postgraduate degree, 31% had a bachelor’s degree, and 11% concluded high school. Most participants worked at the same job more than 10 years (40%), followed by professionals who worked between 2 and 5 years (21%) and between 5 and 10 years (18%). The majority of the participants (93%) were living in southeast Brazil. Most of the employees worked in education services (48%), professional services (37%), and industry (14%) sectors.

### Data analysis

To confirm the original structure of the unifactorial solution of the POCS, confirmatory factor analysis (CFA) was performed. The estimation method used was the weighted least squares mean and variance adjusted (WLSMV) because it is sufficiently robust for ordinal data. The model evaluated the fit indexes of the one-dimension structure in which the 5 items were loaded onto a general perceived opportunities to craft measure (Van Wingerden & Niks, 2017). We also evaluated whether the adapted version of the POCS-L5 showed a unifactorial solution, as with the original structure of the POCS (Van Wingerden & Niks, 2017).

The goodness-of-fit of the POCS-L7 and the POCS-L5 were assessed using the following fit indices: Chi square/degrees of freedom (*χ*^2^/*df*) ratio, the comparative fit index (CFI), the Tucker-Lewis index (TLI), and the root mean square error of approximation (RMSEA). According to the guidelines used, *χ*^2^/*df* should be less than 3, the CFI and TLI values should be greater than 0.95, and the RMSEA values should be less than 0.08 to indicate acceptable fit (with a 90% confidence interval not greater than 0.10). Modification indices higher than 50.00 (MI > 50.00) were also analyzed to identify the sources of any problems in the model specification.

After achieving the most adequate structure of the two versions of the POCS, multigroup confirmatory factor analysis (MGCFA) was performed to test the measurement invariance of the scale for sex. The measurement invariance was evaluated by testing the configural, metric, scalar, and uniqueness invariances in a hierarchical way, which meant that a more restricted model was compared to a less restricted model. The measurement invariance was assessed based on the CFI difference values (ΔCFI) and the RMSEA difference values between the models (ΔRMSEA). Measurement invariance is achieved if ΔCFI is lower than 0.01 (ΔCFI < 0.01) and ΔRMSEA is lower than 0.015 (ΔRMSEA < 0.015) (Putnick & Bornstein, 2016). After confirming that the unidimensional solution was the best structure for the POCS, the reliability of the scale was assessed using ordinal Cronbach’s alpha (α), omega (ϖ), and composite reliability (*cr*).

The evidence based on the relation with external variables was evaluated through the convergent validity and discriminant validity. The convergent validity of the POCS-L7 was assessed using the relations of POCs with occupational self-efficacy, work engagement, job crafting dimensions, and workaholism. The evaluation of the convergent validity of the POCS-L5 was evaluated through the association of POCs with the work engagement and job crafting dimensions. The correlations were investigated through two structural equation models to control the measurement error of the model with one model for each version of the POCS. In order to evaluate the discriminant validity of the two versions of POCS, the values of AVE of each construct and the square correlations of the constructs with each other were calculated. All analysis described in this study were carried out through the software R Studio version 4.0.

### Ethical considerations

The respondents were invited to participate on a voluntary basis. Those individuals who agreed to participate answered the instruments after signing (paper-and-pen data collection) or agreeing with (*online* data collection) the informed consent form. This study received approval from the Ethics Committee of the Institute of Psychology of Salgado de Oliveira University, with CAEE 65103317.6.0000.5289, and from the Ethics Committee of the Pontifical Catholic University of Campinas, with CAEE 23247919.4.0000.548.

## Results

### CFA of POCS-L7 and POCS-L5

The unifactorial solution of the POCS-L7 and that of the POCS-L5 were assessed through two CFAs (WLSMV estimation method). The results of the unifactorial POCS-L7 showed unsatisfactory goodness-of-fit indexes (Table 1).

**Table 1:** Confirmatory factorial analysis of one-dimensional structure for POCS-L7 and POCS-L5

POCS-L7				POCS-L7 (e1-e2 correlated)				POCS-L5			
Items	F. L.	*τ*		Items	F. L.	*τ*		Items	F.L.	*τ*	
1	0.830*	1	− 1.027*	1	0.694*	1	− 0.027*	1	0.795*	1	− 1.346*
		2	− 0.741*			2	− 0.741*			2	− 0.763*
		3	− 0.503*			3	− 0.503*			3	− 0.498*
		4	− 0.176			4	− 0.176			4	0.562*
		5	0.150*			5	0.150*	2	0.651*	1	− 1.264*
		6	0.702*			6	0.702*			2	− 0.735*
2	0.780*	1	− 0.996*	2	0.625*	1	− 0.996*			3	− 0.467*
		2	− 0.619*			2	− 0.619*			4	0.699*
		3	− 0.396*			3	− 0.396*	3	0.579*	1	− 1.833*
		4	− 0.030*			4	− 0.030*			2	− 1.280*
		5	0.357*			5	0.357*			3	− 0.744*
		6	0.934*			6	0.934*			4	0.375*
3	0.729*	1	− 0.438*	3	0.762*	1	− 0.438*	4	0.836*	1	− 1.438*
		2	− 0.112*			2	− 0.112*			2	− 1.047*
		3	− 0.741*			3	− 0.741*			3	− 0.801*
		4	− 0.387*			4	− 0.387*			4	0.236*
		5	0.011*			5	0.011*	5	0.741*	1	− 1.176*
		6	0.522*			6	0.522*			2	− 0.735*
4	0.813*	1	− 0.376*	4	0.851*	1	− 0.376*			3	− 0.294*
		2	− 0.083*			2	− 0.083*			4	0.646*
		3	− 0.845*			3	− 0.845*				
		4	− 0.527*			4	− 0.527*				
		5	− 0.097*			5	− 0.097*				
		6	0.406*			6	0.406*				
5	0.781*	1	− 0.066*	5	0.822*	1	− 0.066*				
		2	− 0.768*			2	− 0.768*				
		3	− 0.517*			3	− 0.517*				
		4	− 0.178*			4	− 0.178*				
		5	0.204*			5	0.204*				
		6	0.679*			6	0.679*				
Goodness-of-fit indexes			*χ*^2^ (*df*)		*χ*^2^/*df*	TLI	CFI	RMSEA (90% C.I.)			
POCS-L7			564.99* (5)		113.00	0.848	0.924	0.321 (0.299–0.343)			
POCS-L7 (e1-e2 correlated)			12.39* (4)		3.10	0.997	0.999	0.044 (0.017–0.073)			
POCS-L5			12.44* (5)		2.48	0.981	0.962	0.064 (0.019–0.110)			

*p < .001

*F. L.* factorial loading, *τ* threshold, *POCS-L7* perceived opportunity to craft scale with seven-point Likert scale, *POCS-L7 (e1-e2 correlated)* perceived opportunity to craft scale with seven-point Likert scale with errors of item 1 and 2 correlated, *POCS-L5* perceived opportunity to craft scale with five-point Likert scale

After analyzing the modification indexes, a second model was evaluated, including the error covariance for item pair 1 and 2 (MI = 226.3). Table 1 shows that the inclusion of the error covariance for item pair 1-2 improved the goodness-of-fit indexes of the unidimensional solution for the POCS-L7, even that the *χ*^2^/*df* remained showing poor values (Table 1). Both items 1 (“At work I have the opportunity to vary the type of tasks I carry out”) and 2 (“At work I have the opportunity to adjust the number of tasks I carry out”) refer to the content and ways of dealing with work demands, respectively. The overlap content of this pair of items might explain the residual correlation.

All items showed high factorial loadings for the unifactorial model of the POCS-L7 with the correlation of the errors of item pair 1 and 2 with values ranging from 0.625 to 0.851. Regarding the thresholds, the scale indicated satisfactory variability in the difficulties of responses. It was observed that the difficulty is greater when the response option of the same item is closer to the “totally agree” alternative, indicating a gradual increase in the response difficulty along the interval scale. The item with greater difficulty of endorsement was item 2 (“At work I have the opportunity to adjust the number of tasks I carry out”).

The CFA results of the POCS-L5 demonstrated that the unifactorial structure had great goodness-of-fit indexes. All items presented high factorial loadings on the unidimensional solution of the POCS, ranging from 0.529 to 0.780 (Table 1). The analysis of the thresholds indicated that the scale showed satisfactory variability in the difficulty of responses. The difficulty of the endorsement of items increased gradually along the interval scale, that is, the difficulty is greater when the response option of the same item is closer to the “totally agree” alternative. The item that participants showed greater difficulty in responding to it in the POCS-L7 and POCS-L5 was item 2 (“At work I have the opportunity to adjust the number of tasks I carry out”), as observed that this item showed the higher threshold (respectively, POCS-L7, *τ =* 0.934, POCS-L5, *τ =* 0.699) (Table 1).

The results suggested that the unidimensional model was the most adequate solution for the Brazilian version of the POCS. Additionally, all item loadings were satisfactory for the two versions of the scale. The threshold values showed that the difficulty of the items being endorsed were similar among the two versions of the POCS (Table 1).

### MGCFA POCS-L7 and POCS-L5

The MGCFA of POCS-L7 evaluated if the unifactorial structure with the correlation of the errors of item pair 1 and 2 was invariant for sex. For POCS-L5, the MGCFA assessed if the unidimensional model was invariant for sex. The goodness-of-fit indexes of the configural model showed that the unidimensional structure of the Brazilian version of the POCS was acceptable for the two versions of the scale according to sex (female and male). The POCS-L7 achieved metric, scalar, and strict invariance according to the ΔCFI of sex (male and female; Table 2). However, according to the ΔRMSEA, the POCS-L7 did not show invariance for sex (male and female; Table 2). Conversely, the POCS-L5 achieved metric, scalar, and uniqueness invariance according to the ΔCFI and ΔRMSEA of sex (male and female; Table 2) (Putnick & Bornstein, 2016).

**Table 2:** Sex (male × female) measurement invariance for POCS-L7 and POCS-L5

POCS-L7 (e1-e2 correlated)	*χ*^2^ (*gl*)	CFI	ΔCFI	RMSEA	ΔRMSEA
Unconstrained model	3.91 (8)	0.999	–	0.038	–
Metric invariance	10.34 (12)	0.998	0.001	0.047	0.010
Scalar invariance	66.32 (36)	0.988	0.010	0.069	0.022
Uniqueness invariance	66.82 (37)	0.995	0.007	0.042	0.027
POCS-L5	*χ*^2^ (*gl*)	CFI	ΔCFI	RMSEA	ΔRMSEA
Unconstrained model	10.06 (10)	0.989	–	0.083	–
Metric invariance	12.64 (14)	0.982	0.007	0.097	0.014
Scalar invariance	30.29 (28)	0.974	0.008	0.086	0.011
Uniqueness invariance	34.86 (29)	0.970	0.004	0.072	0.014

*p < .001

*POCS-L7 (e1-e2 correlated)* perceived opportunity to craft scale with seven-point Likert scale with errors of item 1 and 2 correlated, *POCS-L5* perceived opportunity to craft scale with five-point Likert scale

### Reliability

Reliability coefficients of the POCS-L7 were satisfactory using ordinal Cronbach’s alpha (α (95% C.I.) = 0.878 (0.868–0.888)), the reliability measured by omega (ϖ (95% C.I.) = 0.847 (0.828–0.865)), as well as the composite reliability (*cr* = 0.867). The POCS-L5 also presented satisfactory reliability measured by ordinal Cronbach’s alpha (α (95% C.I.) = 0.784 (0.751–0.814)) by omega (ϖ (95% C.I.) = 0.792 (0.750–0.833), and by the composite reliability (*cr* = 0.846)).

### Evidence based on relation with external variables

It was observed that the AVEs values of all variables were greater than their squared correlations, demonstrating that there was discriminant validity between them. Based on that, the results showed evidence of discriminant validity for the POCS-L7 and POCS-L5. It was observed that POCS-L7 and POCS-L5 differ from overall job crafting, task crafting, cognitive crafting, relational crafting, and work engagement. Furthermore, POCS-L7 obtained discriminant validity evidence with workaholism and occupational self-efficacy.

The convergent validity was evaluated separately for the POCS-L7 and POCS-L5. As expected, the POC levels measured by the POCS-L7 presented significant positive and moderate correlations with occupational self-efficacy, work engagement, overall job crafting, task crafting, cognitive crafting, and relational crafting. POCs were negatively associated with workaholism, with a relation of low magnitude (Table 3). The POCs assessed by the POCS-L5 showed positive and moderate correlations with work engagement, overall job crafting, and job crafting dimensions (i.e., task crafting, cognitive crafting, and relational crafting) (Table 3).

**Table 3:** Means, standard deviation, and correlations

POCS-L7	M (SD)	AVE	1	2	3	4	5	6	7	8
1) POCs	4.1 (1.8)	0.64		0.23	0.25	0.27	0.41	0.30	0.05	0.18
2) CC	3.8 (1.2)	0.82	0.48** [0.39, 0.57]		0.45	0.50	0.74	0.40	0.00	0.20
3) TC	4.0 (1.2)	0.64	0.50** [0.41, 0.58]	0.67** [0.60, 0.73]		0.53	0.74	0.38	0.01	0.26
4) RC	5.0 (1.1)	0.82	0.52** [0.43, 0.60]	0.71** [0.64, 0.76]	0.73** [0.68, 0.78]		0.83	0.45	0.00	0.30
5) JC	4.4 (0.8)	0.64	0.64** [0.57, 0.70]	0.86** [0.82, 0.89]	0.86** [0.83, 0.89]	0.91** [0.88, 0.92]		0.67	0.00	0.41
6) Eng.	4.4 (0.9)	0.82	0.55** [0.46, 0.62]	0.63** [0.56, 0.70]	0.62** [0.54, 0.69]	0.67** [0.61, 0.73]	0.82** [0.78, 0.86]		0.04	0.55
7) Work.	1.9 (0.6)	0.64	− 0.22** [− 0.33, − 0.11]	0.02 [− 0.09, 0.14]	0.08 [− 0.03, 0.20]	0.04 [− 0.08, 0.15]	0.05 [− 0.07, 0.16]	− 0.21** [−0 .32, − 0.10]		0.04
8) OS	4.2 (0.6)	0.82	0.43** [0.33, 0.52]	0.45** [0.35, 0.53]	0.51** [0.42, 0.59]	0.55** [0.47, 0.63]	0.64** [0.57, 0.71]	0.74** [0.69, 0.79]	− 0.20** [− 0.31, − 0.09]	
POCS-L5	*M* (*SD*)	AVE	1	2	3	4	5	6		
1) POCs	3.7 (0.9)	0.81		0.22	0.34	0.19	0.55	0.31		
2) CC	3.6 (0.8)	0.75	0.47** [0.38, 0.55]		0.30	0.24	0.45	0.72		
3) TC	4.1 (0.7)	0.81	0.58** [0.51, 0.65]	0.55** [0.47, 0.62]		0.18	0.56	.27		
4) RC	2.8 (0.6)	0.75	0.44** [0.35, 0.52]	0.49** [0.40, 0.57]	0.42** [0.32, 0.50]		0.41	0.24		
5) JC	3.5 (0.5)	0.81	0.74** [0.68, 0.78]	0.85** [0.81, 0.87]	0.75** [0.70, 0.79]	0.64** [0.58, 0.70]		0.79		
6) Eng.	5.6 (1.1)	0.75	0.56** [0.48, 0.63]	0.67** [0.61, 0.73]	0.52** [0.44, 0.60]	0.49** [0.40, 0.56]	0.89** [0.87, 0.91]			

*p < .05

**p < .001

*POCs* perceived opportunities to craft, *TC* task crafting, *CC* cognitive crafting, *RC* relational crafting, *JC* job crafting, *Eng*. work engagement, *Work*. workaholism, *OS* occupational self-efficacy

The lower diagonal shows the correlations between the latent variables, estimated using structural equations. Values in square brackets indicate the 95% confidence interval for each correlation. In the upper diagonal, the coefficients of determination are presented (that is, the square of the correlation)

## Discussion

The first aim of our study was to adapt the POCS (van Wingerden & Niks, 2017) to the Brazilian context and validate it in order to quantitatively measure POCs. The results of our study provide support for the replication of the unifactorial structure of the POCS-L7 and POCS-L5 in the Brazilian context, supporting our *hypotheses* 1 and 2. Both versions are reliable instruments to access POCs. Nevertheless, the unidimensional model of the POCS-L7 presented a correlation among item pair 1-2, suggesting that some relations were not fully explained by the scale.

The two versions of the Brazilian POCS showed adequate threshold variability. These findings indicate that both versions of the scale evaluate a wide range of POCs, making it possible to differentiate professionals with low and high levels of POCs. Furthermore, in both versions of the POCS, the items presented high factorial loadings. These results corroborate that POCs constitute a unidimensional phenomenon, which encompass professionals’ evaluations about the content of their tasks, as well as their perceptions about the cognitive and relational aspects of their work demands and resources (van Wingerden & Niks, 2017).

The third hypothesis was partially supported. The POCS-L7 did not present full measurement invariance for sex (male and female). However, the POCS-L5 presented full measurement invariance for sex (male and female). Even though female professionals faced gender inequality at work (Qian & Fan, 2019) and reported higher levels of job crafting (Rudolph et al., 2017), we expected that men and women would comprehend POCs in a similar manner. The results indicated that POCS-L5 is an adequate instrument, as it assesses POCs in a similar manner for male and female professionals. POCS-L5 can be used for Brazilian males and females. On the other hand, the lack of measurement invariance of POCS-L7 may hinder meaningful interpretation of the scale between male and female professionals. Future studies can investigate the absence of full measurement invariance for sex in POCS-L7.

Based on the psychometric performance of both POCS-L7 and POCS-L5, regarding the confirmation of unifactorial dimension, goodness-of-fit indexes, measurement invariance for sex, and reliability, we suggest the POCS-L5 may be used in Brazilian psychological research as well as POCS-L7. Some researchers suggest that longer response scales are preferable since they will increase variability in total scores and thus maximize precision and validity; however, no improvements in psychometric precision were identified beyond six response options (Simms et al., 2019). Our results indicated that changing the number of response options from seven-point Likert scale to five-point Likert scale was not detrimental to psychometric performance of POCS. We did not observe any psychometric advantage of POCS-L7 compared to POCS-L5.

It should be noted that some researchers may desire simpler response scales for non-psychometric reasons (e.g., readability, simplicity), yet our results suggest that no additional items are needed to POCS-L5 in order to maintain measurement precision. Additional response scale options may present participants with difficulties to perceive distinct meaning between similarly worded anchor terms (e.g., *strongly agree* vs. *very strongly agree*), and consequently pose challenges in their ability to make decisions regarding their degree of agreement to the items (Simms et al., 2019). Following Brazilian psychological research praxis, we also recommend the use of POCS-L5 with anchor terms in all points of the scale grades (Damásio et al., 2014).

The second goal of this study was to examine the convergent and discriminant validity of the POCS-Brazilian version. POCs were found to be significantly related to job crafting (*hypothesis 4*), occupational self-efficacy (*hypothesis 5*), work engagement (*hypothesis 6*), and workaholism (*hypothesis 7*) in predictable ways. We found that all correlations between POCs and the external variables were positive and moderate, except for a negative relation of low magnitude between POCs and workaholism. These findings provide evidence for the convergent validity of the scale and indicated that POCs and the external variables are theoretically and empirically related yet distinctive constructs.

Regarding the association of POCs and job crafting, a positive relationship of moderate magnitude was found, which gave support to our *hypothesis* 4. POCs were positively related to the three dimensions of job crafting (i.e., task crafting, relational crafting, and cognitive crafting) evaluated with the Brazilian version of the JCQ (Pimenta de Devotto & Machado, 2020; Slemp & Vella-Brodrick, 2013).

We further found evidence that POCs were positively related to approach resources and approach demands crafting (behavioral and cognitive) (*hypothesis 4a*). The JCQ (Slemp & Vella-Brodrick, 2013) evaluated three types of *job crafting content*: approach resources crafting (behavioral), measured with task and relational crafting dimensions; approach demands crafting (behavioral), measured by task crafting dimension); and approach resources crafting (cognitive), measured with cognitive crafting dimension (Zhang & Parker, 2019). The task crafting, relational crafting, and cognitive crafting subscales of the JCQ measured directed and effortful actions to seek positive aspects of work (approach crafting). These JCQ subscales did not assess actions to move away from the negative aspects of work (avoidance crafting). Existing measures of job crafting, including the JCQ (Slemp & Vella-Brodrick, 2013), are limited in their coverage and specific assessment of the whole level of *job crafting content,* as defined in the hierarchical structure of job crafting (Zhang & Parker, 2019). Further studies will need to investigate the associations of POCs and other forms of job crafting content, such as avoidance crafting (behavioral and cognitive).

The POCS-L5 and POCS-L7 were related with overall job crafting and its dimensions, personal resources (i.e., occupational self-efficacy), and occupational well-being indicators (i.e., work engagement and workaholism). The assessment of the discriminant validity of POCS-L5 and POCS-L7 showed that two versions of the instrument evaluated POCs as a unique construct. These findings corroborated *hypothesis* 8, evidencing that POCS-L7 and POCS-L5 showed discriminant validity (Fornell & Larcker, 1981).

The strengths of the study include the robustness of the data analysis procedures. Two versions of the Brazilian adaptation of the POCS were evaluated using two independent samples. Furthermore, all analyses were performed with corrections for the characteristics of ordinal and nonscalar variables.

Our findings support the convergent validity of the POCS by means of correlations with external variables assessed with self-reported measures. However, this type of data does not allow us to make causal inferences and we cannot assert that POCs are a precondition to job crafting. The data collection at one point only is the first limitation of this study. A second limitation regards the fact that our assessment of the relationships of POCs and job crafting content was restricted to the active and positive forms of job crafting. Since we do not have available job crafting measures that cover the avoidance crafting types (i.e., behavioral avoidance resource crafting, behavioral avoidance demand crafting, cognitive avoidance resource crafting, and cognitive avoidance demand crafting) in our context, our assessment of POCs and job crafting was limited to approach crafting.

The third limitation of our study is the use of a nonrepresentative sample. The use of a convenience sampling technique increases the probability that individuals who experience higher levels of POCs or are more willing to craft their jobs and will be more prone to voluntarily collaborate on the research. Future research may examine the causal relationships between POCs and possible antecedents (e.g., occupational self-efficacy, job autonomy, and high demands) and the actual job crafting behavior as a key outcome. It will also be important to investigate the whole range of job crafting content (e.g., approach crafting and avoidance crafting).

## Conclusion

POCs reflect an overall perception of the extent to which employees can influence their jobs and work environments. Employees’ actual job crafting behavior may depend on their perceived opportunities to craft their jobs (Wrzesniewski & Dutton, 2001). Our study adds to the generalizability of the findings by validating the POCS for Brazil. The POCS-Brazilian version is a reliable and valid scale to measure POCs. Researchers can choose to use POCS-L5 with no psychometric disadvantage, when looking for more simplicity and readability of scale grading options for participants. POCs may drive employees’s approach crafting (behavioral and cognitive). POCs are positively related to occupational self-efficacy, work engagement, and negatively related to workaholism.

In a VUCA environment, understanding how to positively influence POCs in order to facilitate job crafting is a matter of importance to organizations and HR from both research and practical perspectives. The perception of not having opportunities to craft may hinder self-initiated changes in job resources and demands, which may prevent employees and organizations of positive outcomes from job crafting. We hope to stimulate new studies to advance the research by assessing POCs as an important variable that influences approach and avoidance crafting.

## Data Availability

The datasets used and/or analyzed during the current study are available from the corresponding author on reasonable request.

## Appendix

Escala de Percepção de Oportunidades para Redesenho do Trabalho—EPORT-L5

(Pimenta de Devotto, Freitas & Wechsler, 2020)

As afirmações a seguir têm por objetivo explorar suas percepções frente às oportunidades que você tem de mudar aspectos do seu trabalho. Por favor, escolha a resposta que melhor se aplica a você, usando a seguinte escala: 1 (discordo totalmente) até 5 (concordo totalmente)

**Table:**

	1 Discordo totalmente	2 Discordo parcial-mente	3 Indife-rente	4 Concordo parcial-mente	5 Concordo totalmente
1. No meu trabalho tenho a oportunidade de variar o tipo de tarefas que desempenho					
2. No meu trabalho tenho a oportunidade de ajustar a quantidade de tarefas que desempenho					
3. No meu trabalho eu tenho a oportunidade de modificar a forma como eu interajo com outras pessoas					
4. No meu trabalho tenho a oportunidade de assumir novas atividades e desafios					
5. No meu trabalho tenho a oportunidade de mudar o significado do meu papel					

## Abbreviations

CFA: Confirmatory factor analysis
CFI: Comparative fit index
CI: Confidence interval
CR: Composite reliability
DUWAS: Dutch Work Addiction Scale
EPORT: Escala de Percepção de Oportunidades para o Redesenho do Trabalho
JCQ: Job Crafting Questionnaire
JD-R: Job demand–resources
MGCFA: Multigroup confirmatory factor analysis
MI: Modification indices
OSS-SF: Occupational Self-efficacy Scale–Short form
POCs: Perceived opportunities to craft
POCS: Perceived opportunities to craft scale
POCS-L7: Perceived opportunity to craft scale with seven-point Likert scale
POCS-L5: Perceived opportunity to craft scale with five-point Likert scale
RMSEA: Root mean square error of approximation
TLI: Tucker-Lewis index
UWES: Utrecht work engagement scale
AVE: Average variance extracted
VUCA: Acronym that stands for volatility, uncertainty, complexity, and ambiguity
WLSMV: Weighted least square mean and variance adjusted
ΔCFI: Difference values of comparative fit index
ΔRMSEA: Difference values of root mean square error of approximation
